# Characterization and Prediction of Mechanical and Chemical Properties of Luanta Fir Wood with Vacuum Hydrothermal Treatment

**DOI:** 10.3390/polym15010147

**Published:** 2022-12-28

**Authors:** Ming-Chi Hsieh, Ke-Chang Hung, Jin-Wei Xu, Yi-Hung Wu, Wen-Shao Chang, Jyh-Horng Wu

**Affiliations:** 1 Department of Forestry, National Chung Hsing University, Taichung 402, Taiwan; 2 Department of Wood Based Materials and Design, National Chiayi University, Chiayi 600, Taiwan; 3 Experimental Forest, National Chung Hsing University, Taichung 402, Taiwan; 4 School of Architecture, University of Sheffield, Sheffield S10 2TN, UK

**Keywords:** luanta fir (*Cunninghamia konishii* Hayata), mechanical properties, cellulose crystallinity, near infrared, predictive modeling, vacuum hydrothermal treatment, non-destructive testing

## Abstract

Since the chemical composition of wood is closely related to its mechanical properties, chemical analysis techniques such as near-infrared (NIR) spectroscopy provide a reasonable non-destructive method for predicting wood strength. In this study, we used NIR spectra with principal component analysis (PCA) to reveal that vacuum hydrothermal (VH) treatment causes degradation of hemicellulose as well as the amorphous region of cellulose, resulting in lower hydroxyl and acetyl group content. These processes increase the crystallinity of the luanta fir wood (*Cunninghamia konishii* Hayata), which, in turn, effectively increases its compressive strength (σ_c,max_), hardness, and modulus of elasticity (MOE). The PCA results also revealed that the primary factors affecting these properties are the hemicellulose content, hydroxyl groups in the cellulose amorphous region, the wood moisture content, and the relative lignin content. Moreover, the ratios of performance deviation (RPDs) for the σ_c,max_, shear strength (σ_s,max_), hardness, and modulus of rupture (MOR) models were 1.49, 1.24, 1.13, and 2.39, indicating that these models can be used for wood grading (1.0 < RPD < 2.5). Accordingly, NIR can serve as a useful tool for predicting the mechanical properties of VH-treated wood.

## 1. Introduction

The global focus on the research and development of sustainable resources has drawn attention to wood due to its renewability, environmental friendliness, and carbon sequestration ability. Wood, a biological and organic material which is susceptible to biotic (e.g., bacteria, fungi, and termites) and abiotic hazards (e.g., ultraviolet light, thermal stress, and desiccation), is prone to deformation, warping, splitting, decay, discoloration, and degradation during long-term outdoor use. Consequently, some studies have attempted to use thermal modification to extend the lifespan of wood materials [1,2,3]. To this end, four heat treatment media are commonly used: air [4,5], inert gas or vacuum [6], oil [7], and steam [8,9,10]. Of these, air treatment lowers the degradation temperatures of cellulose, hemicellulose, and lignin and increases their degradation rate because of the presence of ambient oxygen, which reacts with hydroxyl groups to produce oxidized functional groups, such as carbonyls, carboxyls, and hydroperoxides [4]. Conversely, steam treatment produces hydronium ions (H_3_O^+^), which accelerate the hydrolysis of polysaccharides but also reduce oxidation. Steam can also retard the hydrolysis of cellulose at lower temperatures [8,9]. Furthermore, in dry heat treatment, wood must be dried in advance to avoid the cracking caused by rapid water evaporation during the heat treatment. By contrast, in closed-system treatments, moisture can evaporate from the wood or be supplemented externally; thus, steam can be used to both control the relative humidity inside the reaction tank to reduce cracking and to protect against oxidation. Furthermore, the H_3_O^+^ produced by steam accelerates the hydrolysis of hydrophilic polysaccharides, and steam can soften the noncrystalline polymers inside the wood cell walls, increasing their reactivity at low temperatures (compared with that in a dry environment) [11,12]. Vacuum hydrothermal (VH) treatment is mainly used to accelerate the degradation of hydrophilic polysaccharides by leveraging the H_3_O^+^ produced by the autoionization of water at high temperatures as a catalyst in low-oxygen environments. In addition to effectively reducing the occurrence of cracks in wood caused by rapid heating, VH treatment also lowers treatment energy consumption [13]. Luanta fir (*Cunninghamia konishii* Hayata), a tree species endemic to Taiwan, is an outcrossing, long-lived conifer [14]. The wood and leaf oils of luanta fir possess activity against the larvae of *Aedes aegypti* and *A. albopictus* [15]. In addition, luanta fir wood is advantageous for its straight texture and lightness; historically, it was one of the most widely used wood construction and building materials in Taiwan. In our previous study, luanta fir wood was modified through VH treatments under various conditions, and a prediction model for its physical properties was created using a nondestructive spectroscopy technique [16]. This study is a continuation of that research; in it, we mainly explored the impact of different conditions of VH treatment on the mechanical and chemical properties of luanta fir wood.

The hemicellulose present in wood is degraded during heat treatment, producing formic acid, acetic acid, and other degradation products. These acidic products further degrade the amorphous regions of cellulose, which may reduce the mechanical strength of the wood [3]. Wentzel et al. [17] modifed *Eucalyptus nitens* wood with 100% relative humidity (RH) VH treatment and reported that such treatments at 160 °C reduced the modulus of elasticity (MOE) and modulus of rupture (MOR) of the wood from 18 GPa and 119 MPa to 15 GPa and 89 MPa, respectively. This reduction may have been due to the cracking, collapse, and deformation of the wood cell walls during the heat treatment [18]. Moreover, Wentzel et al. [19] also reported that *Eucalyptus nitens* wood treated with high RH VH developed cracks in the middle lamella. Additionally, fiber separation and pore generation occurred, enabling water to enter the cell walls, thereby affecting the water absorption and mechanical properties of the wood. According to Willems [20], using steam caused the test material to reach hygroscopic equilibrium with its surroundings, reducing fiber hornification during treatment and the internal stress caused by high-temperature drying. Furthermore, wood that was hydrothermally treated in a humid environment softened and gained increased molecular chain mobility, leading to the rearrangement of amorphous-region molecules and decreased crystallization activation energy, resulting in increased crystallinity and improved mechanical properties. Therefore, in addition to testing the effects of various VH treatment conditions (time and temperature) on the mechanical properties of wood, the effects of these different conditions on the wood microstructures, crystallinity, and chemical structures were also observed in this study.

Conventional heat treatments of wood are typically performed with heat conduction; thus, the degree of heat treatment may vary within the same batch of wood in the treatment tank. The degree of treatment affects the dimensional stability, mechanical strength, and durability of the heat-treated material, which, in turn, affects subsequent applications. Consequently, a simple and rapid method of assessing the properties of heat-treated materials is necessary for quality control. Many scholars have used spectral analysis methods, such as visible spectroscopy, middle-infrared spectroscopy, and near-infrared spectroscopy (NIRS), for non-destructive and rapid assessments or predictions of the properties of wood materials [21,22,23]. Of these, NIRS has been widely used to identify wood types, detect defects, and predict the physical and mechanical properties of wood [22,24,25,26,27,28,29]. Therefore, in addition to exploring the effects of various VH conditions on the mechanical properties and chemical structures of luanta fir wood, this study also aimed to establish an NIRS model for predicting the mechanical properties of heat-treated wood.

## 2. Materials and Methods

### 2.1. Materials

The flat-sawn luanta fir wood used in this study was purchased from Jang Chang Lumber Industry Co., Ltd. (Hsinchu, Taiwan). The specimens were 600 mm long × 138 mm wide × 26 mm thick. Before VH treatment, all specimens were conditioned at 20 °C and 65% relative humidity (RH) for at least 2 weeks, and a total of 352 specimens that demonstrated an MOE of between 5.0 and 6.5 GPa under a load of 10 N were selected for subsequent tests to reduce the influence of individual variations on the specimen properties.

### 2.2. Vacuum Hydrothermal Treatment of Luanta Fir Wood

A semi-industrial reactor equipped with a thermocouple and pressure sensors (San Neng Ltd., Chiayi, Taiwan) was used for the VH treatment of luanta fir wood. The wood and a proper amount of water were placed in the tank without coming into contact with each other, and a vacuum pump was used to lower the internal pressure to <23 kPa. Next, the temperature was increased to 160, 180, 200, 220, or 240 °C at a rate of 3 °C/min. After reaching the target temperature, heat treatments were carried out for 4, 8, or 16 h (these temperature ranges and durations are frequently used for wood heat treatment). The specimens were then cooled to room temperature before undergoing moisture conditioning at 20 °C with 65% RH in preparation for subsequent experiments.

### 2.3. Determination of Mechanical Properties

To determine the mechanical properties of untreated and VH-treated luanta fir wood, several determinations, including longitudinal maximum compressive strength (σ_c,max_), shear strength (σ_s,max_), cross-sectional hardness, and flexural properties, were conducted by a universal testing machine (Shimadzu AG-10kNX, Tokyo, Japan). Briefly, the σ_c,max_, σ_s,max_, and hardness of specimens with dimensions of 40 mm × 20 mm × 20 mm, 30 mm × 20 mm × 20 mm, and 20 mm × 20 mm × 20 mm were measured with a loading speed of 5 mm/min according to the Chinese National Standards, i.e., CNS 453 [30], CNS 455 [31], and CNS 460 [32], respectively. The σ_c,max_ (MPa) was calculated using Equation (1):

σ_c,max_ (MPa) = P/A (1)

where P is the maximum load (N) and A is the cross-sectional area (mm^2^) of the specimen. The σ_s,max_ (MPa) was calculated using Equation (2):

σ_s,max_ (MPa) = V/b_s_h_s_ (2)

where V is the maximum applied force (N), b_s_ is width (mm) of the shear plane, and h_s_ is the length (mm) of the shear plane. The hardness (N) was calculated using Equation (3):

Hardness (N) = P/10(3)

where P is the load (N) when a 10-mm-diameter steel ball was pressed 0.32 mm deep into the specimen surface. The flexural properties were determined according to CNS 454 [33]. The MOR and MOE of the specimens with dimensions of 600 mm × 138 mm × 26 mm were determined through a three-point flexural test at a loading speed of 5 mm/min and a span of 364 mm. The MOR, MOE, MOR retention ratio, and MOE retention ratio were calculated using Equation (4), Equation (5), Equation (6), and Equation (7), respectively:

MOR (MPa) = 3*PL*/2*bh*^2^ (4)

MOE (MPa) = Δ*PL*^3^/4Δ*Ybh*^3^ (5)

MOR retention ratio (%) = (MOR_h_/MOR_u_) × 100 (6)

MOE retention ratio (%) = (MOE_h_/MOE_u_) × 100 (7)

where *P* is the maximum load (N), *L* is the length (mm) of the span, Δ*P* is the difference of load (N) between 10 % and 40 % values of the maximum load, ∆*Y* is deflection (mm) due to ∆*P*, *b* is the width (mm) of the specimen, and *h* is the height (mm) of the specimen. MOR_h_ and MOE_h_ are the MOR and MOE, respectively, of the VH-treated materials. MOR_u_ and MOE_u_ are the MOR and MOE of the untreated specimens. All samples were conditioned at 20 °C and 65% RH for two weeks prior to testing, and 15 replicate specimens were tested for each group.

### 2.4. Scanning Electron Microscope Imaging

A JEOL JSM-7800F (Tokyo, Japan) scanning electron microscope (SEM) was used to observe the surface of the various heat-treated specimens. After sputter coating the specimens with platinum, surface morphological images of samples were captured by SEM at an accelerating voltage of 3.0 kV.

### 2.5. X-ray Diffraction Analysis

A Bruker D8 SSS (Leipzig, Germany) high-resolution X-ray diffractometer was used to analyze the effects of different heat treatment conditions on specimen crystallinity. The diffraction patterns were measured from 2*θ* = 2° to 35° using CuKα1 radiation at 40 kV and 30 mA. One specimen was used for each measurement. Peak separations were measured using the PeakFit deconvolution software (Systat Software, Richmond, CA, USA), and all *R*^2^ values were greater than 0.990. The crystallinity index (CrI) of the samples was calculated using Equation (8) [34]:

CrI (%) = [*A*_cry_/(*A*_cry_ + *A*_am_)] × 100 (8)

where *A*_cry_ is sum of the crystalline band areas (i.e., the 101, $10\bar{1}$, and 002 lattice reflections of the cellulose crystallographic form at 2*θ* = 14°, 16°, and 22°, respectively) and *A*_am_ is the area of the amorphous band at 2*θ* = 18°.

### 2.6. Solid-State CP/MAS ^13^C NMR Analysis

The samples were examined by solid-state cross polarization/magic angle spin (CP/MAS) carbon-13 nuclear magnetic resonance (^13^C NMR). The spectra were recorded on a Bruker DSX-400WB FTNMR spectrometer (Bremen, Germany) with a sampling frequency of 100 MHz. The chemical shifts were calculated relative to tetramethylsilane (TMS).

### 2.7. NIR Spectral Measurements

A PerkinElmer Spectrum Two N (Buckinghamshire, UK) NIR spectrometer was used to determine functional group changes on the surfaces of the untreated and VH-treated specimens. The spectra were collected by co-adding 32 scans at a resolution of 4 cm^−1^ in the 4000 to 10,000 cm^−1^ range.

### 2.8. Constructing the Property Prediction Models

The PerkinElmer Spectrum 10 software (Buckinghamshire, UK) with spectrum quant advanced algorithms pack (ver. 10.6.2.1159) was used to analyze the mechanical properties and NIR spectra of the VH-treated specimens and to construct the property prediction models. For each property, 240 sets of data were obtained from the untreated group and the 15 VH treatment groups (15 replicates in each group); 80% of the data were used as the calibration set, and the remaining 20% were used as the prediction set. Spectral preprocessing comprised normalization (i.e., multiplicative signal correction (MSC) and standard normal variate (SNV)), baseline correction, derivative, and Savitzky–Galay (SG) filtering. Polynomial smoothing functions with 5 points and 49 points were used for the first and second derivative with SG filtering, respectively. The number of latent variables (LVs) was set to 10, and model building was performed with partial least squares regression (PLSR). In addition, cross-validation (leave-one-out technique) was used in all cases for calibration model evaluation. The coefficient of determination for calibration (*R*^2^_c_), root mean squared error (RMSE) of calibration (RMSEC), and RMSE of cross-validation (RMSECV) were used to identify the optimal preprocessing conditions. The LVs were set to 1–10 to identify the optimal LVs and eliminate the 10 data that did not conform with the model. The final prediction model was constructed with the same preprocessing conditions and LVs. Finally, the prediction sets were input to the model for verification. The accuracy of the model was verified by calculating the coefficient of determination for prediction (*R*^2^_p_), the RMSE of prediction (RMSEP), and the ratio of performance deviation (RPD) with measured data from the same set.

### 2.9. Analysis of Variance

All results are expressed in terms of mean ± standard deviation (SD). The significance of differences was calculated by Scheffe’s test or Student’s *t*-test, and *p* values < 0.05 were considered to be significant.

## 3. Results and Discussions

### 3.1. Effects of VH Treatment Conditions on the Mechanical Properties of Luanta Fir Wood

Typically, long, high-temperature heat treatment improves the dimensional stability, durability, and decay resistance of wood. However, the degradation products of such treatments accelerate the degradation of the wood and reduce its mechanical properties. Therefore, to understand how the VH treatment conditions affect the mechanical properties of luanta fir wood, the σ_c,max_, σ_s,max_, hardness, and flexural properties of VH-treated luanta fir wood were tested.

Table 1 reveals that untreated luanta fir wood has a σ_c,max_ of 38 MPa. Except for the specimens treated at 240 °C for 8 or 16 h, the other heat-treated specimens showed some increase in σ_c,max_. A Student’s *t*-test revealed significant differences in σ_c,max_ between the untreated group and the VH-treated specimens at 160 °C/4 h, 160 °C/16 h, 180 °C/4 h, 180 °C/8 h, 200 °C for 4 to 16 h, 220 °C/4 h, and 240 °C/4 h; these groups had σ_c,max_ of 43–45 MPa, indicating that the σ_c,max_ of luanta fir wood increased after heat treatment. This increase was attributed to the lower water content, increased crystallinity, and greater rigidity (due to the cross-linking properties of lignin) of the heat-treated wood [35]. However, the 240 °C/8 h and 240 °C/16 h specimens had lower σ_c,max_ than the untreated specimens, i.e., 30.7 MPa and 17.4 MPa, respectively,. These conditions might have caused the degradation of the hemicellulose and lignin, increasing the reactive surface area of the cellulose and lowering the cellulose degradation temperature, reducing the cellulose crystallinity, simultaneously leading to defibration and defibrillation [36,37]. Therefore, under appropriate treatment conditions, VH treatment can improve the σ_c,max_ of luanta fir wood. As for the shear strength, the σ_s,max_ of untreated luanta fir wood is 9.5 MPa. VH treatment at 160–220 °C had no significant effects on the σ_s,max_ of luanta fir wood; all specimens had σ_s,max_ of 8.5–10.9 MPa (Table 1). However, the 240 °C/8 h and 16 h specimens had drastically reduced σ_s,max_, i.e., 4.6 and 4.1 MPa, respectively—less than half that of the untreated group. Heat treatment at high temperatures (240 °C) for a long period of time (> 8 h) might have lowered the degree of polymerization (DP) in the cellulose and hemicellulose, increasing the brittleness of the wood; moreover, cracks could have appeared in the secondary wall and compound middle lamella of the cell walls, reducing the σ_s,max_ [38,39,40,41]. Additionally, as presented in Table 1, untreated luanta fir wood has a cross-sectional hardness of 29 N. The hardness of treated luanta fir wood specimens increased with temperature and duration, except for the 240 °C/16 h specimens, for which hardness significantly decreased to 19 N. Only the 200 °C/8 h, 220 °C/4 h, and 240 °C/16 h specimens had significantly different hardness from the untreated specimens. Among them, the hardness of the 200 °C/8 h specimens and 220 °C/4 h specimens increased significantly to 35 N. This might have been due to the increased crystallinity that resulted from these conditions, which would increase the surface hardness of the wood. However, the cellulose crystalline region begins to degrade at higher temperatures and longer durations [42], lowering crystallinity and reducing hardness.

On the other hand, Table 1 reveals that untreated luanta fir wood has an MOE of 5.7 GPa. The MOE first decreased and then increased as the VH treatment temperature and duration increased. For the 160 °C specimens, those with 4-h treatment had the highest MOE (6.3 GPa). At treatment times of 8 or 16 h, the specimens treated at 200 °C had the highest MOE (6.7 and 6.2 GPa, respectively), which was significantly greater than that of the untreated specimens. A similar trend can be observed in MOE retention, which was highest among the 200 °C/8 h specimens (118%). This was attributed to the initial degradation of hemicellulose during heat treatment, leading to a decrease in the flexural strength of the wood [35]. At the higher treatment temperature of 200 °C, the increasing crystallinity and decreasing equilibrium moisture content again increased the rigidity of the wood [43]. However, at a treatment temperature of 240 °C, the cellulose crystalline region begins to degrade, lowering the air-dry density and the rigidity of the wood [35].

Moreover, the MOR of the untreated luanta fir wood was 58 MPa. In the treated specimens, although the MOR decreased as treatment temperature and duration increased, differences among the groups were typically nonsignificant; the MOR was 40–59 MPa and MOR retention was ≥70% among almost all treated specimens. However, the MOR of the 240 °C/8 h and 16 h specimens decreased considerably to 8 and 3 MPa, respectively; the MOR retention was 14% and 5%, respectively

### 3.2. Effects of VH Treatment Conditions on the Chemical Structure of Luanta Fir Wood

**Figure 1:**
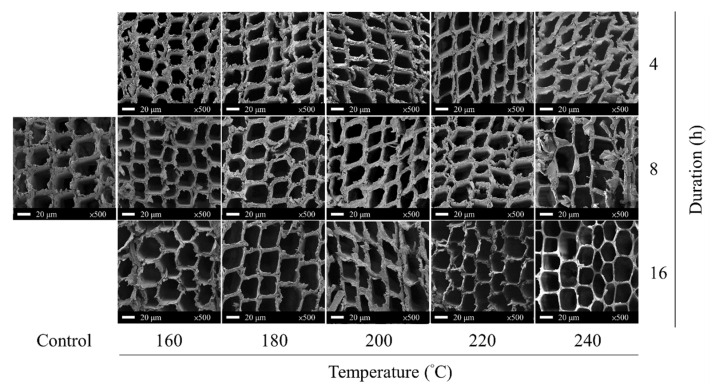
Cross-sectional SEM of untreated (control) and VH-treated luanta fir wood.

Heat treatment changes the microstructure of wood; therefore, SEM was used to observe the tracheids of earlywood in the specimens. The results are presented in Figure 1. As shown, after the VH treatments, the 160 °C/4 h and 160 °C/8 h SEM images were similar to those of the untreated specimens. Further increases in treatment temperature and duration resulted in the occurrence of delamination cracks in the middle lamella and a decrease in cell wall thickness. For the 240 °C/16 h specimens, the secondary wall practically vanished, supporting the hypothesis that this treatment would reduce the mechanical properties of the wood. Generally speaking, cell walls in wood comprise the middle lamella, primary wall, and secondary wall. The middle lamella and primary wall contain more lignin than the secondary wall and are less likely to degrade during heat treatment. Conversely, the secondary wall contains more hemicellulose and is consequently the first component to degrade during heat treatment, leading to the thinning of the cell walls. The dissipation of bound water in the wood and degradation of cellulose during heat treatment are additional factors contributing to the decreased thickness of the cell walls [44,45,46].

**Figure 2:**
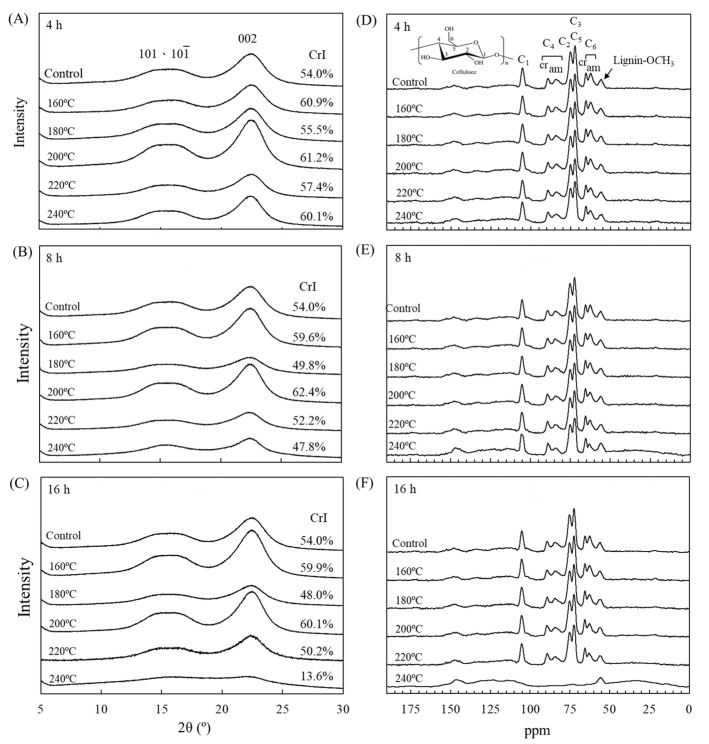
XRD (**A**–**C**) and NMR (**D**–**F**) spectra of luanta fir wood before and after VH treatment at different temperatures for (**A**,**D**) 4, (**B**,**E**) 8, and (**C**,**F**) 16 h.

To examine correlations among crystallinity, water absorption, and mechanical properties, X-ray diffraction (XRD) was used to detect crystal structures in each group of specimens and to calculate the crystallinity. The results in Figure 2A–C reveal that the untreated luanta fir wood had visible diffraction peaks at scanning angles (2*θ*) of 15° and 22°, which represent the diffraction peaks of the cellulose 101/$10\bar{1}$ and 002 crystal planes. VH treatment did not result in a significant displacement of the two diffraction peaks. The untreated luanta fir wood had a CrI of 54.0%. Therefore, VH treatment at 160 °C did increase the crystallinity. This was attributed to the dehydration and condensation of the cellulose molecular chains producing ether bonds during heat treatment at 160 °C, resulting in the orderly arrangement of the amorphous-region microfibrils and slight increases in crystallinity [47]. However, at 180 °C for 8 and 16 h, the CrI of the heat-treated specimens decreased to 49.8% and 48.0%, respectively. This was attributed to the DP of lignin decreasing during treatment at 180 °C, resulting in plasticization, which increases the abundance of hydrophilic groups and the extent of the cellulose amorphous region [48]. Conversely, crystallinity was observed to increase at treatment temperatures of 200 °C; heat treatment at 200 °C/8 h resulted in the highest crystallinity (CrI = 62.4%). The inferred cause is that during heat treatments at 200 °C, the xylan and galactose in the hemicellulose amorphous region degraded into small molecules, resulting in relative increases in crystallinity. Furthermore, these small-molecule degradation products aggregated and recrystallized over time [47]. However, if the treatment temperature was further increased to 240 °C, treatment for 8 or 16 h caused the CrI to drop to 47.8% and 13.6%, respectively; this was mainly due to the degradation of the cellulose crystalline region [47,48]. These changes are consistent with MOE trends: the 200 °C/8 h specimens had the highest MOE retention ratio (118%) and CrI (62.4%), whereas the 240 °C/16 h specimens had the lowest (11% and 13.6%, respectively).

The chemical structures of the VH-treated specimens were analyzed using solid-state CP/MAS ^13^C NMR. The ^13^C NMR maps all underwent normalization with the C_1_ absorption peak at 105 ppm; the results are depicted in Figure 2D–F. In the untreated luanta fir wood, numerous characteristic absorption peaks of cellulose appeared in the 60–120 ppm range: the C_4_ characteristic absorption peaks in the crystalline and amorphous regions at 90 and 85 ppm, respectively; the C_2_ characteristic absorption peak at 75 ppm; the C_3_ and C_5_ characteristic absorption peak at 72 ppm; and the C_6_ characteristic absorption peaks in the crystalline and amorphous regions at 65 and 62 ppm, respectively. Moreover, the peaks at 120–150 ppm and 56 ppm are the absorption peaks of lignin benzene rings and methoxy groups, respectively. Among the VH-treated specimens, the intensity of the cellulose C_4_ and C_6_ absorption peaks in the amorphous region—at 85 ppm and 62 ppm, respectively—decreased as the treatment temperature and duration increased, indicating that VH treatment of luanta fir wood leads to the degradation of the cellulose amorphous region and a relative increase in crystallinity. At the same time, increased treatment temperature and duration led to the intensification and shift of the lignin characteristic absorption peaks, revealing that lignin structural changes occurred as a result of condensation and cross-linking [49,50]. Furthermore, the 240 °C/8 h specimens had some decreases in the C_4_ and C_6_ absorption peaks in the cellulose amorphous region at 90 ppm and 65 ppm, whereas the cellulose characteristic absorption peaks in the 60–120 ppm range practically vanished in the specimens treated at 240 °C/16 h. This indicates that cellulose crystalline regions began degrading at 240 °C/8 h and almost completely deteriorated by 16 h, which is consistent with the XRD results.

**Figure 3:**
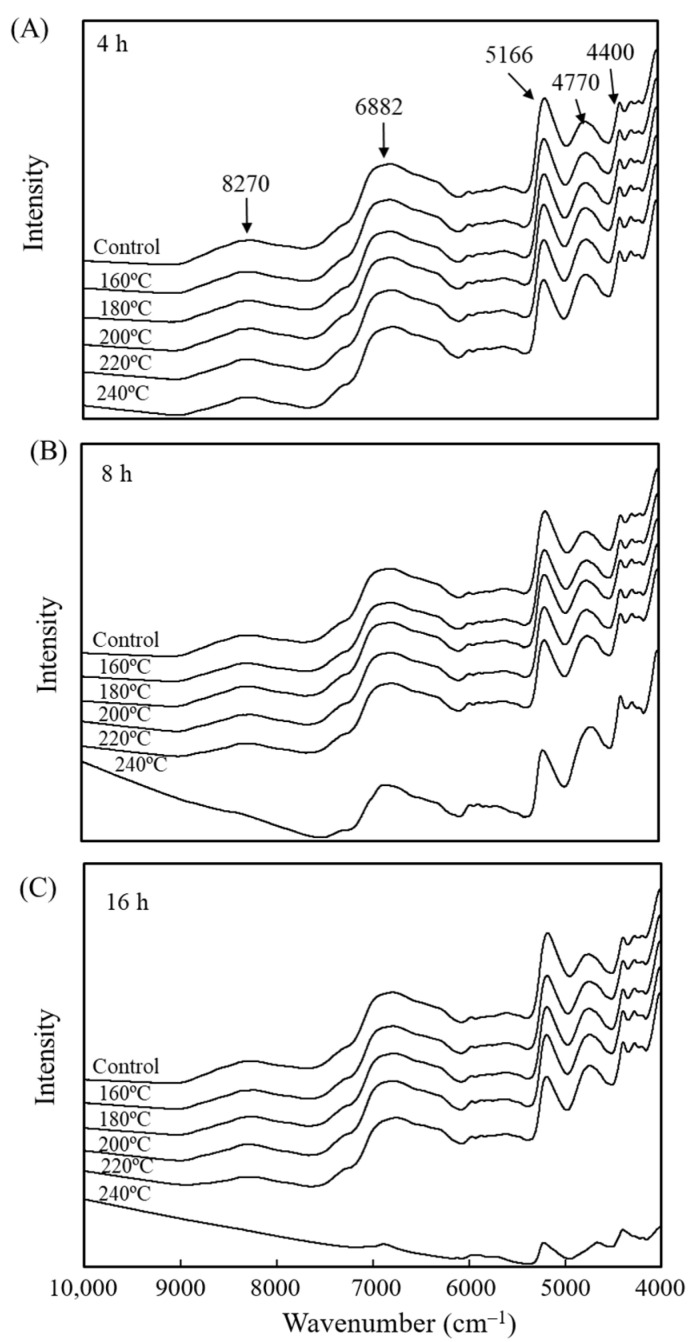
NIR spectra of luanta fir wood before and after VH treatment at different temperatures for (**A**) 4, (**B**) 8, and (**C**) 16 h.

To further understand the effects of various VH treatment conditions on the surface chemical functional groups of luanta fir wood, each group of specimens was tested using NIRS; the results are presented in Figure 3. According to Popescu et al. [51], wood NIR spectra can be divided into five zones: wavenumbers 9000–7500 cm^−1^ are the first and second overtone bending vibration absorbances of the C–H functional groups within the carbohydrates and lignin methoxy and methylene groups; wavenumbers 7500–6100 cm^−1^ are the first overtone absorption band of the C–H groups and the first overtone stretching vibration absorbance of the O–H groups; wavenumbers 6100–5400 cm^−1^ are the stretching vibrations of the C–H groups in the aliphatic and aromatic compounds and the vibration absorbances of the O–H groups in all the main components; wavenumbers 5400–4525 cm^−1^ are the stretching and deformation vibration absorption peaks of the C=O groups, O–H groups, and aromatic compounds; and wavenumbers 4525–4000 cm^−1^ are the stretching and deformation vibration absorbance of the C–H groups. As revealed in Figure 3, the untreated luanta fir wood exhibited visible absorption peaks in five zones: at 8270, 6882, 5166, 4770, and 4400 cm^−1^.

**Figure 4:**
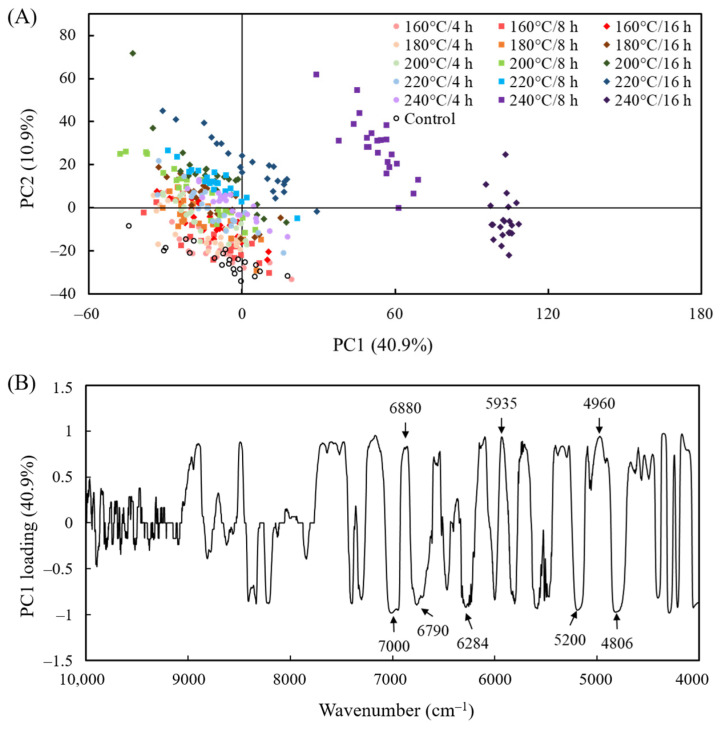
PCA scatter plot for the (**A**) NIR spectrum and (**B**) variable loading of PC1 for untreated and VH-treated luanta fir wood.

However, because the functional groups of each main component had highly overlapping absorption bands in the NIR spectra, the differences in functional groups between the treated specimens and untreated specimens were not apparent. Therefore, the NIR spectra were further analyzed using principal component analysis (PCA). Figure 4A presents a PC1 to PC2 scatter plot of the NIR spectra of the untreated and VH-treated luanta fir wood specimens. Specimens with different heat treatments could not be clearly distinguished by PC1; only those treated at 240 °C/8 h and 240 °C/16 h were distinct. Conversely, specimens could be roughly distinguished with PC2. The untreated specimens generally had negative PC2 values; these values generally became more positive as the heat treatment temperature and duration increased. However, 240 °C/8 h and 16 h specimens were indistinguishable from other treated specimens with PC2. Furthermore, the PC1 variable loading shown in Figure 4B reveals that the factors of positive PC1 values were lignin characteristic peaks at 6880, 5935, and 4960 cm^−1^, whereas those for negative PC1 values were absorption peaks associated with the hydroxyl groups in cellulose, hemicellulose, and water molecules at 7000, 6790, 6284, 5200, and 4806 cm^−1^. These analysis results indicate that the 240 °C/8 h and 16 h specimens could be discerned using PC1 mainly because they contained more lignin and less cellulose and hemicellulose than the other treated specimens [22,52,53]. The inferred reason is that the degradation and repolymerization of lignin can occur simultaneously in an aqueous medium. In the initial reaction stage, breaking the bonds between lignins and carbohydrates led to the degradation of phenylpropane units and other lignin molecules with low molecular weight and high reactivity; condensation and cross-linking reactions occur as organic acids are produced, leading to the formation of insoluble products that increase the relative lignin content. The wood cell walls become thinner as the lignin and hemicellulose degrade, increasing the reactive surface area of the cellulose and lowering the temperature at which cellulose begins to degrade. This, in turn, lowers the relative cellulose content [36,54,55,56].

### 3.3. Mechanical Property Prediction Model for VH-Treated Luanta Fir Wood Based on NIRS

NIRS is a simple and efficient technique that has been used in chemical composition analysis in many fields [57]. In this study, PLSR was used to match NIRS with the mechanical properties of the wood to obtain a high-accuracy predictive model. The model was constructed with the NIR spectra, σ_c,max_, σ_s,max_, cross-sectional hardness, MOE, and MOR of 240 specimens of luanta fir wood treated at different temperatures and durations. In this study, 192 sets of data (80%) were used as the calibration set; the remaining 48 sets (20%) were used as the prediction set. The value ranges for each property are presented in Table 2.

Furthermore, due to the high levels of overlap and the presence of numerous collinearities and irrelevant information in the NIR spectra, the absorption bands of the principal component functional groups could not be easily distinguished, which affected the reliability and robustness of the model. Therefore, to facilitate subsequent analysis and model building, the spectra were preprocessed to strengthen the signals and reduce noise [58]. Preprocessing comprised multiplicative signal corrections, standard normal variates, baseline corrections, derivative, and SG filtering. Subsequently, *R*^2^_c_ and RMSEC were calculated with 10 LVs; the results are presented in Table 3. Generally speaking, a high *R*^2^_c_ value and a low RMSEC value are ideal. Table 3 reveals that multiplicative signal corrections, standard normal variates, and baseline corrections did not lead to significant differences in *R*^2^_c_ or RMSEC; however, the first and second derivative processing increased the *R*^2^_c_ and decreased the RMSEC. Using σ_c,max_ as an example, *R*^2^_c_ increased from 0.500 to 0.810 and 0.737, whereas the RMSEC decreased from 6.75 to 4.16 and 4.89; this was attributed to the ability of derivatives to remove both additive and multiplicative effects in the spectra [59]. The first derivative removes only the baseline, while the second derivative removes both the baseline and the linear trend. Furthermore, spectra that underwent second differentiation and SG filtering had the highest *R*^2^_c_ and lowest RMSEC values. The *R*^2^_c_ values for all characteristics were ≥0.9; thus, the second derivative with SG filtering effectively increased the smoothness of the spectrum and reduced noise interference [60,61]. These results are consistent with findings of Sofianto et al. [62]; therefore, these preprocessing conditions were applied for the subsequent tests.

Furthermore, to prevent data overfitting and increase the reproducibility of the model (i.e., a model with fewer LVs and lower RMSEC values), the methodology of Mitic et al. [63] was referenced, and the number of LVs with a difference of less than 20% between the RMSEC and the RMSECV and with the smallest RMSEC value was chosen as the optimal number. Table 4 presents the results of building PLSR predictive models with various numbers of LVs. Accordingly, the optimal number of LVs for the σ_c,max_, σ_s,max_, hardness, MOE, and MOR predictive models were 4, 4, 5, 6, and 5, respectively.

**Figure 5:**
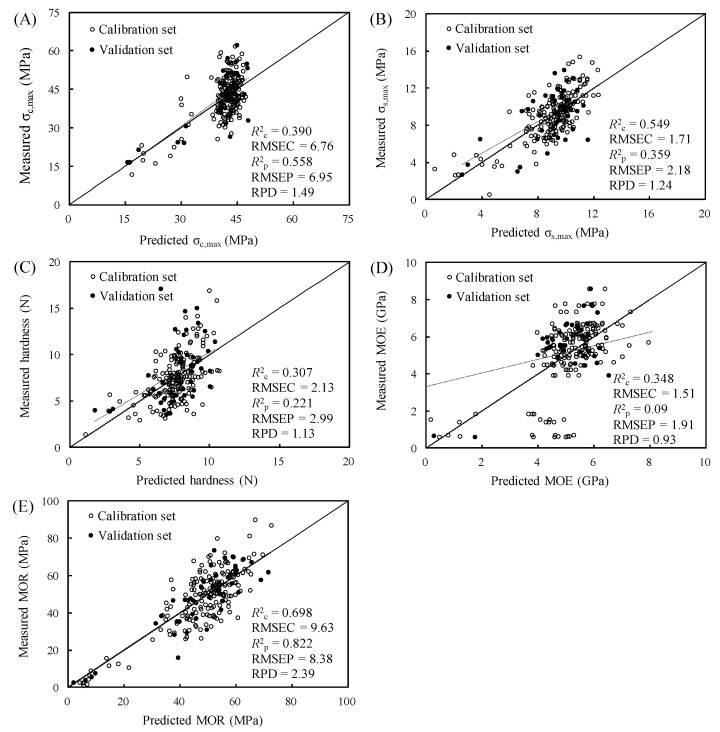
Correlation between the predicted and measured values of (**A**) σ_c,max_, (**B**) σ_s,max_, (**C**) hardness, (**D**) MOE, and (**E**) MOR.

The predictive models of the mechanical properties of the study specimens were constructed according to these conditions. To test the accuracy of the predictive models, the prediction sets were input to the models to obtain the coefficient of determination for prediction (*R*^2^_p_), the RMSEP, and the RPD; the accuracy of the test results is depicted in Figure 5. The results indicated that when linear regression through the origin was performed for each characteristic, the MOR predictive model had the highest *R*^2^_c_ value (0.698), whereas the hardness predictive model had the lowest (0.307); thus, the MOR model had the best modeling power. However, the σ_s,max_, hardness, and MOE models had substantially decreased *R*^2^_p_ compared with *R*^2^_c_, indicating poor predictive performance. Furthermore, the RMSEP of these models were greater than the RMSEC, indicating poor stability. Conversely, the MOR model had a greater *R*^2^_p_ (0.822) than *R*^2^_c_ (0.698), and its RMSEP (8.38) was significantly lower than its RMSEC (9.63), indicating favorable predictive power. Furthermore, RPD is the ratio of the SD of the measured values within the prediction set to the RMSEP; this value can be used to assess the accuracy of the model. Higher values indicate greater accuracy. If RPD = 1.0–2.5, the model can be used for wood grading [64,65,66]. Figure 5 reveals that the RPDs of the σ_c,max_, σ_s,max_, hardness, and MOR models were 1.49, 1.24, 1.13, and 2.39, i.e., all between 1.0 and 2.5; thus, these four models could be used for grading. The MOR model had an *R*^2^_p_ of 0.822 and an RPD of 2.39, the highest among the models, and the regression lines of its predicted and measured values had slopes near 1, indicating that it had the best predictive power. By contrast, the MOE model had the lowest *R*^2^_p_ value and RPD, i.e., 0.089 and 0.93, respectively; ergo, this model was not suitable for predictions [67,68].

**Figure 6:**
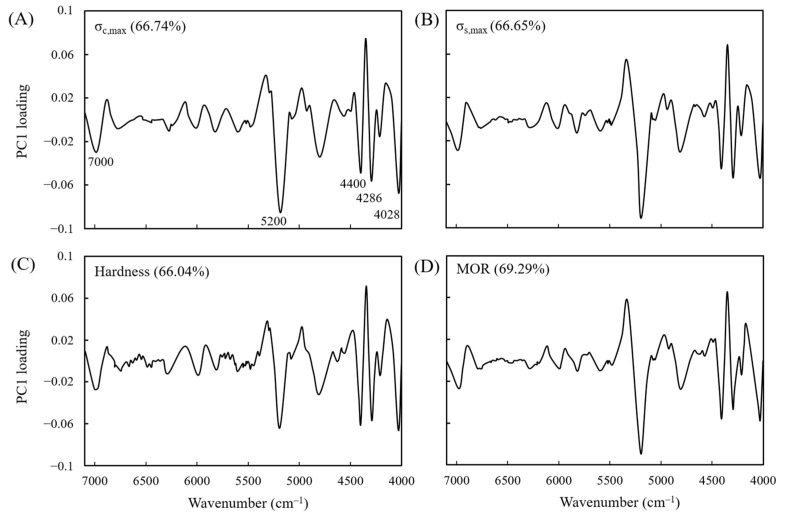
PLSR loading vectors for the (**A**) σ_c,max_, (**B**) σ_s,max_, (**C**) hardness, and (**D**) MOR in the prediction models.

To further understand the main factors affecting the predictive models with RPDs of 1.0–2.5, the PC1 variable loading of the NIR predictive model was further examined. The analysis results are depicted in Figure 6. The main characteristic peaks in the graphs are the absorption peaks of hydroxyl groups in the cellulose amorphous region at wavenumber 7000 cm^−1^, of hydroxyl groups in water molecules at 5200 cm^−1^, of C–H vibrations in cellulose and hemicellulose at 4400 cm^−1^, of hemicellulose at 4286 cm^−1^, and of lignin at 4028 cm^−1^. These results indicate that changes in the hemicellulose content, in the hydroxyl groups in the cellulose amorphous region, in the moisture content of the wood, and in the relative lignin content are the main factors contributing to changes in the mechanical properties of VH-treated wood. Kobori et al. [69] also asserted that water and cellulose are the major factors of a wood properties prediction model.

## 4. Conclusions

Luanta fir wood was subjected to VH treatment at temperatures of 160–240 °C for 4–16 h. In addition to examining the mechanical properties of the study specimens, PCA was performed to determine the factors differentiating VH-treated specimens. A mechanical properties predictive model was also built based on NIRS. The results demonstrate that, in terms of mechanical properties, following VH treatment at 160 °C/8 h, 180 °C/8 h, 200 °C/8 h, 200 °C/16 h, 220 °C/4 h, and 220 °C/8 h, the luanta fir wood retained ≥80% of its flexural strength. However, if the VH treatment temperature was increased to 240 °C and the duration was extended to 8 or 16 h, the mechanical properties of the wood decreased considerably. Furthermore, the XRD, solid-state ^13^C NMR, and NIR results demonstrated that VH treatment of luanta fir wood caused the hemicellulose and cellulose amorphous regions to degrade. Additionally, the hemicellulose underwent deacetylation and dehydration reactions that lowered the hydroxyl and acetyl group contents, thereby increasing the crystallinity and dimensional stability of luanta fir wood. Moreover, at 240 °C, the cellulose crystalline region began to degrade after 8 h of treatment and was practically completely degraded after 16 h. During the heat degradation process, the methoxy groups and aliphatic side chains in lignin broke and then underwent condensation and cross-linking reactions with the acidic degradation products of polysaccharides, increasing the relative lignin content. The PCA results further verified that the untreated and mildly treated specimens contained relatively more cellulose and hemicellulose, whereas the highly treated specimens contained more lignin. Furthermore, the NIR-based predictive model of the mechanical properties of VH-treated luanta fir wood was found to be applicable to the grade screening of MOR, σ_c,max_, σ_s,max_, and hardness (1.0 < RPD < 2.5); the MOR model had the best predictive power. Additionally, the result of PC1 variable loading revealed that the main factors affecting the mechanical properties of VH-treated wood were the hemicellulose content, hydroxyl groups in the cellulose amorphous region, the wood moisture content, and the relative lignin content. These results suggested that an NIR spectrometer could serve as a useful instrument for the prediction of the mechanical properties or for controlling the quality of VH-treated wood. 

## Figures and Tables

**Table 1:** Compressive strength (σ_c,max_), shear strength (σ_s,max_), hardness, MOE, and MOR of untreated and VH-treated luanta fir wood.

Sample	σ_c,max_ (MPa)	σ_s,max_ (MPa)	Hardness (N)	MOE (GPa)	MOE Retention (%)	MOR (MPa)	MOR Retention (%)
Control	38 ± 4 ^ab^	9 ± 3 ^a^	29 ± 5 ^ab^	5.7 ± 0.6 ^abc^	-	58 ± 10 ^ab^	-
160 °C/4 h	45 ± 7 ^a,^ *	10 ± 2 ^a, ns^	28 ± 6 ^ab, ns^	6.3 ± 0.6 ^ab,^ *	110 ± 10 ^ab^	59 ± 16 ^ab, ns^	102 ± 28 ^a^
160 °C/8 h	41 ± 7 ^ab, ns^	10 ± 3 ^a, ns^	28 ± 5 ^ab, ns^	5.8 ± 0.6 ^abc, ns^	102 ± 10 ^abc^	59 ± 8 ^a, ns^	103 ± 13 ^a^
160 °C/16 h	44 ± 6 ^a,^ *	9 ± 2 ^a, ns^	30 ± 7 ^ab, ns^	5.5 ± 0.5 ^bcd, ns^	97 ± 9 ^bcd^	57 ± 13 ^ab, ns^	99 ± 23 ^ab^
180 °C/4 h	44 ± 6 ^a,^ *	11 ± 2 ^a, ns^	30 ± 8 ^ab, ns^	5.3 ± 0.5 ^bcd,^ *	92 ± 8 ^bcd^	55 ± 9 ^ab, ns^	95 ± 16 ^ab^
180 °C/8 h	43 ± 6 ^ab,^ *	9 ± 2 ^a, ns^	28 ± 5 ^ab, ns^	4.7 ± 0.5 ^cd,^ ***	83 ± 8 ^cd^	48 ± 9 ^ab,^ *	83 ± 15 ^ab^
180 °C/16 h	43 ± 10 ^ab, ns^	8 ± 2 ^ab, ns^	28 ± 6 ^ab, ns^	4.6 ± 0.4 ^d,^ ***	80 ± 8 ^d^	42 ± 8 ^ab,^ ***	73 ± 13 ^ab^
200 °C/4 h	44 ± 7 ^a,^ *	10 ± 2 ^a, ns^	30 ± 7 ^ab, ns^	5.4 ± 1.1 ^bcd, ns^	94 ± 19 ^bcd^	58 ± 10 ^ab, ns^	100 ± 17 ^ab^
200 °C/8 h	45 ± 7 ^a,^ *	10 ± 2 ^a, ns^	35 ± 7 ^a,^ *	6.7 ± 0.9 ^a,^ **	118 ± 15 ^a^	57 ± 11 ^ab, ns^	98 ± 20 ^ab^
200 °C/16 h	44 ± 5 ^a,^ *	9 ± 2 ^a, ns^	32 ± 7 ^a, ns^	6.2 ± 0.6 ^ab,^ *	108 ± 11 ^ab^	46 ± 9 ^ab,^ *	79 ± 15 ^ab^
220 °C/4 h	46 ± 8 ^a,^ *	10 ± 2 ^a, ns^	35 ± 8 ^a,^ *	6.1 ± 0.7 ^ab, ns^	107 ± 12 ^ab^	52 ± 10 ^ab, ns^	91 ± 17 ^ab^
220 °C/8 h	41 ± 6 ^ab, ns^	9 ± 2 ^a, ns^	32 ± 7 ^a, ns^	5.9 ± 0.6 ^ab, ns^	103 ± 10 ^ab^	44 ± 12 ^ab,^ *	78 ± 21 ^ab^
220 °C/16 h	42 ± 8 ^ab, ns^	9 ± 3 ^a, ns^	33 ± 6 ^a, ns^	5.6 ± 0.6 ^abcd, ns^	98 ± 10 ^bcd^	40 ± 15 ^b,^ **	69 ± 27 ^b^
240 °C/4 h	43 ± 7 ^ab,^ *	8 ± 2 ^ab, ns^	29 ± 7 ^ab, ns^	5.9 ± 0.6 ^ab, ns^	103 ± 11 ^ab^	43 ± 10 ^ab,^ **	75 ± 17 ^ab^
240 °C/8 h	31 ± 8 ^b,^ *	5 ± 2 ^bc,^ ***	31 ± 6 ^a, ns^	1.7 ± 0.3 ^e,^ ***	29 ± 5 ^e^	8 ± 4 ^c,^ ***	14 ± 6 ^c^
240 °C/16 h	17 ± 3 ^c,^ ***	4 ± 1 ^c,^ ***	19 ± 4 ^b,^ *	0.6 ± 0.2 ^e,^ ***	11 ± 3 ^e^	3 ± 1 ^c,^ ***	5 ± 2 ^c^

Values are mean ± SD (*n* = 15). Columns with different letters indicate significant differences (*p* < 0.05). ns: nonsignificant; *: *p* < 0.05; **: *p* < 0.01; ***: *p* < 0.001 (one-tailed test) compared with control.

**Table 2:** Ranges of mechanical properties of the calibration and prediction sets in the NIR analysis model.

Properties	Calibration Data				Prediction Data			
	Min	Max	Mean	N	Min	Max	Mean	N
σ_c,max_ (MPa)	12	64	41	192	17	62	43	48
σ_s,max_ (MPa)	1	16	9	192	3	14	9	48
Hardness (N)	1	17	7	192	4	17	8	48
MOE (GPa)	0.1	8.6	5.2	192	0.6	8.6	5.2	48
MOR (MPa)	1	90	46	192	2	74	45	48

N: number of sample data.

**Table 3:** PLSR model results with different preprocessing methods.

Properties	Preprocessing Method	LVs	*R* ^2^_c_	RMSEC
σ_c,max_	Original spectra	10	0.500	6.75
	MSC	10	0.515	6.65
	SNV	10	0.522	6.60
	MSC + baseline correction	10	0.515	6.64
	1stDer	10	0.810	4.16
	1stDer + SG	10	0.773	4.55
	2ndDer	10	0.737	4.89
	2ndDer + SG	10	0.949	2.20
σ_s,max_	Original spectra	10	0.482	2.03
	MSC	10	0.481	2.04
	SNV	10	0.508	1.98
	MSC + baseline correction	10	0.486	2.03
	1stDer	10	0.832	1.16
	1stDer + SG	10	0.752	1.41
	2ndDer	10	0.750	1.41
	2ndDer + SG	10	0.947	0.65
Hardness	Original spectra	10	0.304	2.21
	MSC	10	0.309	2.21
	SNV	10	0.332	2.17
	MSC + baseline correction	10	0.307	2.21
	1stDer	10	0.773	1.27
	1stDer + SG	10	0.659	1.55
	2ndDer	10	0.623	1.63
	2ndDer + SG	10	0.928	0.72
MOE	Original spectra	10	0.793	0.77
	MSC	10	0.810	0.74
	SNV	10	0.824	0.71
	MSC + baseline correction	10	0.816	0.73
	1stDer	10	0.936	0.43
	1stDer + SG	10	0.923	0.47
	2ndDer	10	0.916	0.49
	2ndDer + SG	10	0.977	0.26
MOR	Original spectra	10	0.694	10.73
	MSC	10	0.691	10.79
	SNV	10	0.703	10.57
	MSC + baseline correction	10	0.691	10.79
	1stDer	10	0.904	6.03
	1stDer + SG	10	0.868	7.05
	2ndDer	10	0.873	6.91
	2ndDer + SG	10	0.973	3.21

**Table 4:** PLSR model results with different LVs.

Properties	LVs	*R* ^2^_c_	RMSEC	RMSECV	Difference (%)
σ_c,max_	1	0.363	7.58	7.61	0
	2	0.451	7.06	7.15	1
	3	0.466	7.01	7.12	2
	4	0.490	6.92	7.14	3
	5	0.575	6.43	7.78	21
	6	0.672	5.64	7.96	41
	7	0.761	4.94	8.11	64
	8	0.858	3.78	8.10	114
	9	0.910	2.98	8.23	176
	10	0.949	2.25	8.24	266
σ_s,max_	1	0.330	2.27	2.28	1
	2	0.413	2.14	2.16	1
	3	0.436	2.11	2.17	3
	4	0.456	2.08	2.17	4
	5	0.562	1.89	2.27	20
	6	0.665	1.66	2.32	40
	7	0.758	1.44	2.33	62
	8	0.847	1.13	2.30	104
	9	0.915	0.87	2.29	163
	10	0.947	0.68	2.28	236
Hardness	1	0.099	2.47	2.49	0
	2	0.131	2.45	2.48	1
	3	0.177	2.40	2.47	3
	4	0.205	2.37	2.46	4
	5	0.306	2.22	2.53	14
	6	0.442	2.02	2.70	33
	7	0.616	1.84	2.78	52
	8	0.779	1.37	2.79	103
	9	0.866	1.01	2.79	176
	10	0.928	0.75	2.78	271
MOE	1	0.581	1.08	1.09	1
	2	0.693	0.93	0.95	1
	3	0.726	0.89	0.91	3
	4	0.763	0.83	0.85	4
	5	0.853	0.66	0.77	16
	6	0.877	0.61	0.72	18
	7	0.924	0.48	0.71	49
	8	0.943	0.43	0.70	65
	9	0.963	0.33	0.72	115
	10	0.977	0.27	0.72	170
MOR	1	0.554	12.74	12.81	1
	2	0.666	11.08	11.17	1
	3	0.694	10.65	10.98	3
	4	0.704	10.51	10.87	3
	5	0.733	10.06	10.92	9
	6	0.791	9.15	11.75	28
	7	0.864	7.23	11.79	63
	8	0.921	5.59	11.73	110
	9	0.954	4.40	11.56	162
	10	0.973	3.36	11.34	238

## Data Availability

The data presented in this study are available on request from the corresponding author.
